# Coevolution of diagenetic fronts and fluid-fracture pathways

**DOI:** 10.1038/s41598-022-13186-1

**Published:** 2022-06-03

**Authors:** Ardiansyah Koeshidayatullah, Nawwar Al-Sinawi, Peter K. Swart, Adrian Boyce, Jonathan Redfern, Cathy Hollis

**Affiliations:** 1 Present Address: Department of Geosciences, College of Petroleum Engineering and Geosciences, King Fahd University of Petroleum and Minerals, Dhahran, 31261 Saudi Arabia; 2 School of Natural Sciences, The University of Manchester, Manchester, M13 9PL UK; 3 Rosenstiel School of Marine and Atmospheric Sciences, University of Miami, Miami, 33149 USA; 4 Scottish Universities Environmental Research Centre, Glasgow, G75 0QF UK

**Keywords:** Geochemistry, Geology, Mineralogy, Sedimentology

## Abstract

Diagenetic boundaries are paleo-reaction fronts, which have the potential to archive the termination of metasomatic processes in sedimentary rocks. They have not been extensively studied, perhaps because they appear simple in outcrop. Recent work has demonstrated the significance of paleo-reaction fronts to decipher multiphase recrystallization processes and provide high porosity zones. This paper provides a detailed documentation of reaction front evolution in a tectonically active salt basin and reveals a high level of complexity, associated with multiple fluid flow and tectonic events. Here, consistent patterns of increasing dolomite stoichiometry and ordering, along with a change from seawater-derived, fabric-retentive dolomite to fracture-controlled, fabric-destructive hydrothermal dolomite are observed vertically across the stratabound dolomite bodies. These patterns, coupled with a decrease in porosity, increase in ∆_47_ temperature and δ^18^O_water_ values indicate multiphase recrystallization and stabilization by warm, Mg-rich fluids. The stratabound dolomite bodies apparently terminated at a fracture-bound contact, but the presence of dolomite fragments within the fracture corridor suggests that fracturing post-dated the first dolomitization event. The termination of dolomite formation is therefore interpreted to be associated with a decrease in the capacity of the magnesium-rich fluids to dolomitize the rock, as indicated by the presence of non-stoichiometric and poorly ordered dolomite at the reaction fronts. The fracture corridors are interpreted to exploit dolostone-limestone boundaries, forming prior to a later, higher temperature, hydrothermal dolomitization event, which coincided with the formation and growth of the anticline. Karstification subsequently exploited these fracture corridors, widening fractures and leading to localized collapse and brecciation. The results demonstrate that an apparently simple reaction front can have a complex history, governed by the inheritance of prior diagenetic events. These events modified rock properties in such a way that fluid flow was repeatedly focused along the original dolomite-limestone boundary, overprinting much of its original signature. These findings have implications to the prediction of structurally controlled diagenetic processes and the exploration of naturally fractured carbonate reservoirs for energy exploration globally.

## Introduction

Dolomitization is the most common metasomatic process in the carbonate rock record and has been widely studied^1,2^. It occurs by the transformation of calcite (CaCO_3_) to stoichiometric dolomite (CaMg(CO_3_)_2_) and is regarded as a porosity-enhancing process based on either negative volume change (i.e. replacement of larger calcite crystal with smaller dolomite crystal) or mole per mole replacement following reaction (1)^2^:

1 $${\text{2CaCO}}_{{3}} {\text{(s)}} + {\text{Mg}}^{{{2} + }} {\text{(aq)}} - {\text{CaMg }}\left( {{\text{CO}}_{{3}} } \right)_{{2}} {\text{(s)}} + {\text{Ca}}^{{{2} + }} {\text{(aq)}}$$

**Figure 1:**
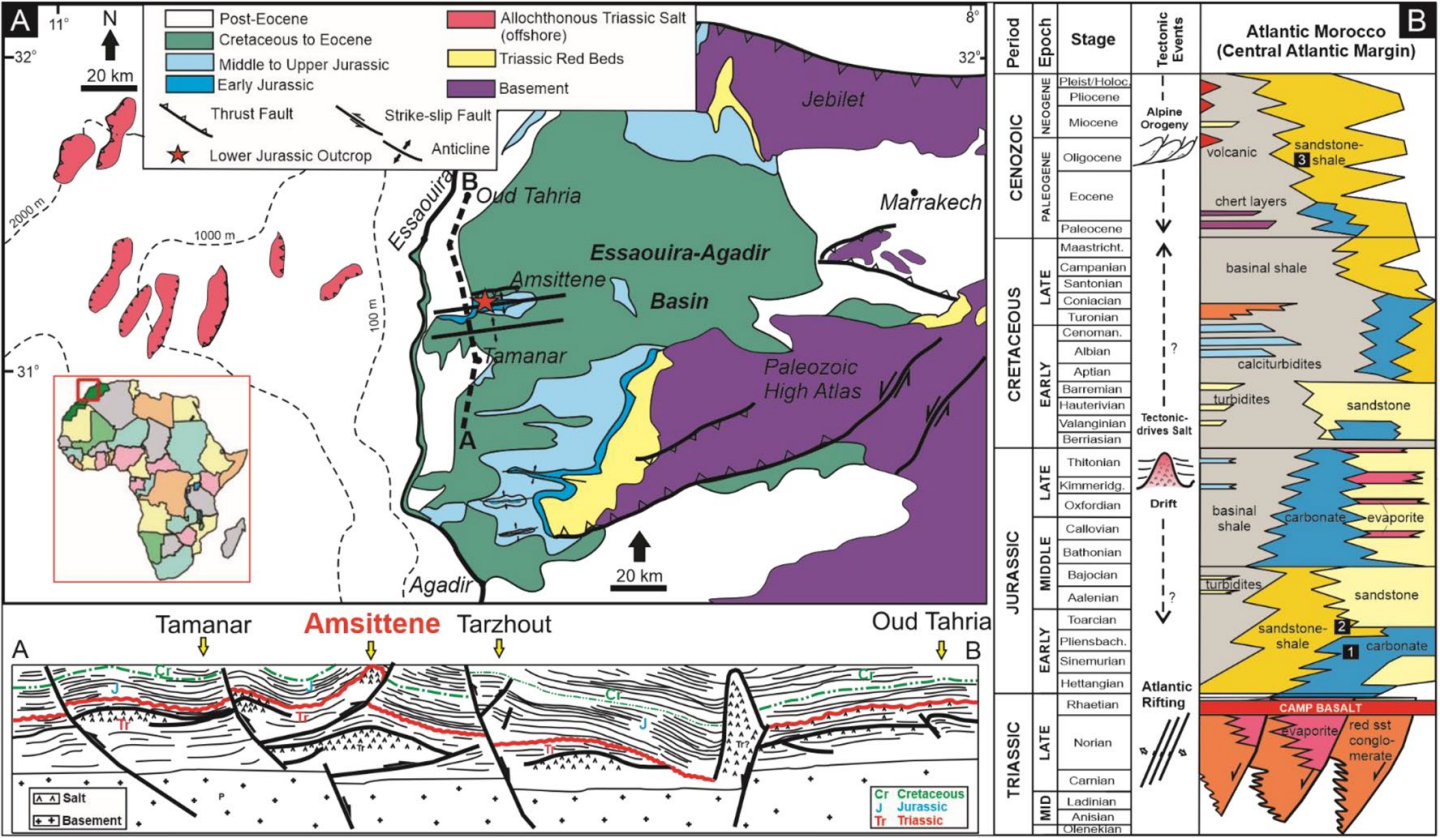
(**A**) Geological map of the Western High Atlas showing the Essaouira-Agadir basin (EAB) onshore and its extension offshore^14,20^. The study location is highlighted with a red star (Amsittene Anticline). The lower image depicts the cross section from A-B which shows the overall basin structure and influence of salt diapir as well as deep transfer fault to the formation of Amsittene anticlinal structure (Hafid, 2000). (**B**) Generalized stratigraphy of the Essaouira-Agadir basin (EAB) and the corresponding tectonic events^11,20^. The numbered black rectangles represent: 1. Arich Ouzla Formation, 2. Amsittene Formation, and 3. Im-n-Tanout Formation.

Dolomitization can lead to the formation of distinct dolomite bodies which can influence the storage capacity and heterogeneity of hydrocarbon, ore and CO_2_ storage reservoirs. These bodies can have distinct dolomite–limestone reaction fronts^2–5^. Previous studies have demonstrated that these reaction fronts can control the spatial variability of porosity^5,6^ and the accumulation of ore minerals^7,8^. Nevertheless, the controls on their occurrence and position have received remarkably little attention. This study is based on a Lower Jurassic carbonate platform that outcrops in part in the salt-cored Amsittene Anticline, Essaouira-Agadir Basin (EAB), Western Morocco (Fig. 1A). It provides a unique opportunity to map dolomite body geometries and their associated dolomitization fronts in a single location in order to: (1) decipher the controls on the position of dolomitization fronts; (2) understand the potential control of salt-related deformation to dolomitization; (3) explore the relationship between reaction fronts, mechanical deformation and diagenesis in a partially dolomitized carbonate platform.

## Geological framework

The Essaouira–Agadir Basin (EAB) is located in south-western Morocco (Fig. 1). During the Early to Late Jurassic, the western margin of Morocco was a passive margin, and carbonate deposition in the basin took place in a subtropical to arid climate^9,10^. Carbonate sedimentation was interrupted periodically by the incursion of clastic sediments from the hinterland to the east, leading to a stacked succession of carbonate platforms separated by sandstone and mudrock^11^ (Fig. 1B). The first carbonate platform occurs in the Arich Ouzla Formation which outcrops solely in the Amsittene Anticline^12,13^ (Fig. 1A, B). The Amsittene Anticline is situated in the northern part of the EAB (Fig. 1A) and cored by a diapir of Triassic salt, which is interpreted to be associated with an E-W trending Late Triassic-Early Jurassic Tarzhout-Ihchech transfer fault^14^ (Fig. 1A). The Arich Ouzla Formation is dated as Upper Sinemurian to Lower Pliensbachian based on the abundant presence of Spiriferina^15,16^. This formation is unconformably overlain by conglomeratic, red, fluvial sandstones of the Amsittene Formation^11^ (Fig. 1B). The maximum burial depth of the Lower Jurassic strata in the northern part of the EAB, particularly in the Amsittene Anticline was very shallow (≤ 1.5 km)^11^ and the geothermal gradient in this basin is around 25–30 °C/km^17,18^.

The development and evolution of the EAB was influenced by various stress regimes, from Mesozoic rifting and salt tectonism to Cenozoic orogenic deformation^14,19,20^. Faults with a N–S orientation formed during the opening of the Atlantic Ocean in the Late Triassic while E–W and NE–SW anticlines formed by Triassic salt tectonic movements during the Jurassic to Cretaceous^14,20^ (Fig. 1A, B). A later NE-SW fault trend, possibly re-activating older Variscan structures, reflects a compressional fault system established during the Alpine Orogeny^21^.

## Methods

A total of 37 carbonate samples were collected from two logged stratigraphic sections and other selected positions within the dolomite bodies. Fracture density was measured using several perpendicular scan lines. Samples were described petrographically using transmitted light microscope and a CITL Mk5 Cold cathodoluminescence stage. Computer-based thin section porosity analysis was conducted on five images for each thin section. Dolomite stoichiometry and ordering were calculated from the X-ray diffraction (XRD) pattern by using Bruker D8Advance Diffractometer following this equation NCaCO_3_ = 333.33d-911.00^22^ and ratio between 015/0110 peaks^23^, respectively. For stable carbon (δ^13^C), oxygen (δ^18^O) and clumped isotopes (Δ_47_) analyses, thin section counterparts were micro-drilled under a binocular microscope to extract different diagenetic phases and limestone matrix. The δ^13^C and δ^18^O analysis were conducted at the Scottish Environmental University Research Centre (SUERC), Glasgow by using a VG OPTIMA mass spectrometer (Isoprime Limited, Manchester, UK). The value for oxygen was corrected by applying a carbonate-phosphoric acid fractionation factor of 1.0008 for both calcite and dolomite^24^, as indicated from previous works^25,26^. All values are reported as delta values with respect to the Vienna PeeDee Belemnite (VPDB) and standardized to Carrara marble and NBS-19. Average analytical precision was ± 0.2‰ for both δ^18^O_VPDB_ and δ^13^C_VPDB_.

For the clumped isotopes, the samples were analysed using a dual inlet Thermo Fisher Scientific 253 and 253 + ultra-high-resolution isotope ratio mass spectrometers following the methodology proposed by earlier work^26,27^. To ensure accuracy, three replicate measurements were made (see supplementary material). All data are reported using the Carbon Dioxide Equilibrated Scale (CDES)^28^. The temperature of formation was calculated from Δ_47_ values using an equation derived from 11 carbonates precpitated at temperatures ranging between 5 and 75 °C and reacted at 90 °C, with no application of an acid fractionation factor^26^.

$$\Delta \_47\textperthousand = 0.0392\left( {0.0017} \right)*10^{ \wedge } 6 / T^{ \wedge } 2 + 0.158\left( {0.018} \right)$$

All the clumped isotopes results are reported as ‰ and with their mean ± Standard Deviation (SD). Different fractionation calibration equations were used to calculate the parent fluid δ^18^O composition of (1) calcite^29^, (2) high temperature dolomite^30^, and low temperature dolomite^31^. The results are reported relative to Standard Mean Ocean Water (δ^18^O_SMOW_).

## Results

### Dolomite, reaction fronts and fractures

**Figure 2:**
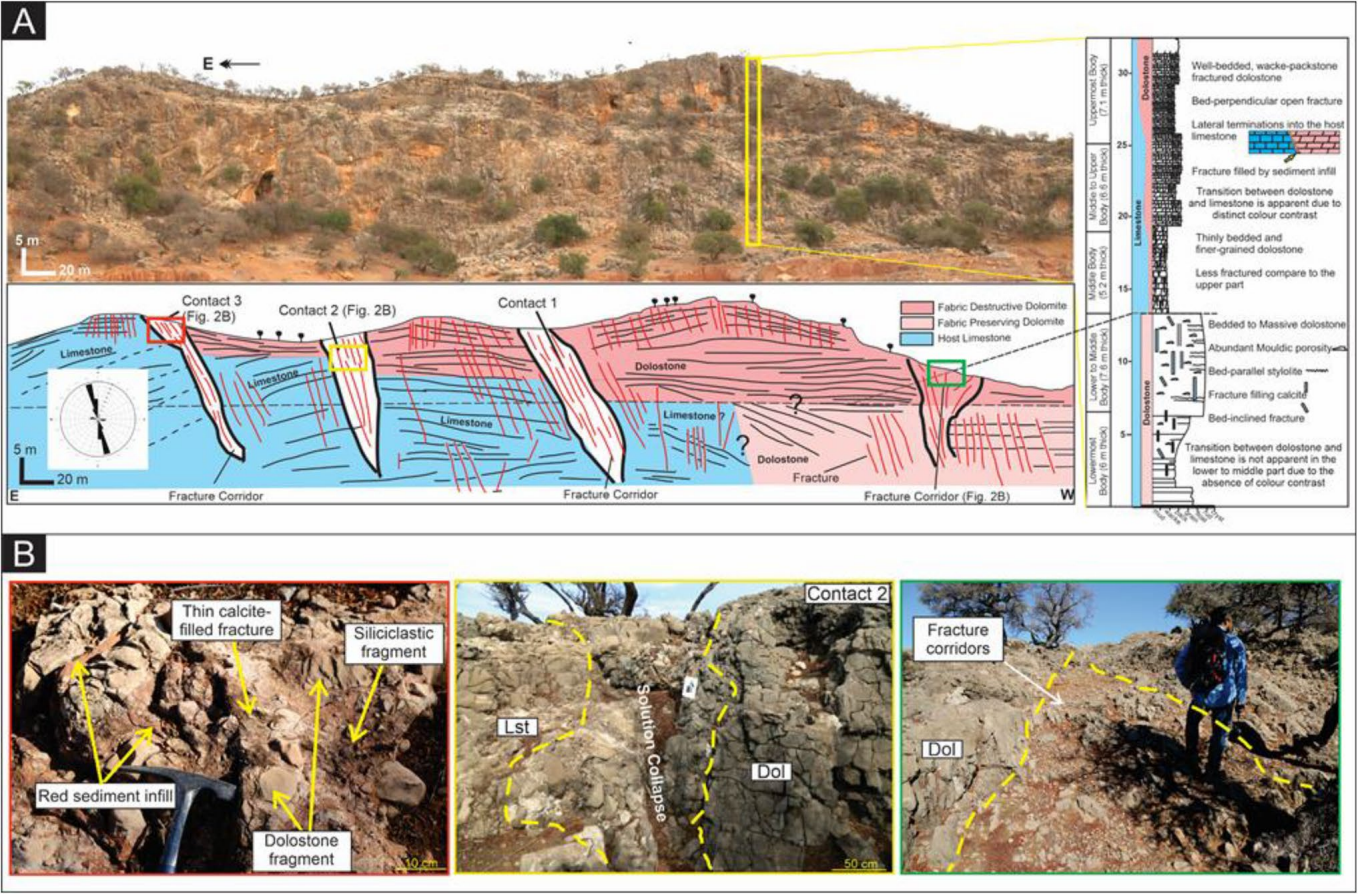
(**A**) Panoramic photos of the studied Early Jurassic outcrop in the Amsittene Anticline. Note the sketch displays several fracture corridors associated with dolomite-limestone contact. The right panel shows a measured sedimentary section of the Lower Jurassic carbonate. (**B**) Left panel: variation of filling material within fracture corridor, including calcite cement, rock fragments (carbonate and siliciclastic) and red sediment infill. Middle panel: dolomite (Dol)-limestone (Lst) bounded by wide deformation zone in contact 2. Right panel: highly fractured dolomite (Dol) and deformation bands within fracture corridors.

The study area is a laterally continuous carbonate section up to 32.5 m in thickness, with a series of stratabound terminations to a more extensive dolomite body that can be sub-divided into five intervals that become wider and thicker up-section (Fig. 2A, B):

(i) Lowermost interval (6 m thick, up to 95–120 m wide) of fabric-preserving (FP) dolomite within well-bedded ooidal grainstone facies,
(ii) Lower to middle interval of FP dolomite (7.6 m thick, up to 120 m wide) within a massive bed of ooidal-oncoidal grainstone to packstone,
(iii) Middle interval of fabric-destructive (FD), bed-parallel, tabular dolomite (5.2 m thick, 152 m wide) within bioclastic wackestone to packstone beds,
(iv) Middle to upper interval (6.6 m thick, 178 m wide) of FD dolomite within bioclastic wackestone,
(v) Upper interval (7.1 m thick, 214 m wide) body of FD dolomite within thinly bedded bioclastic mudstone.

**Figure 3:**
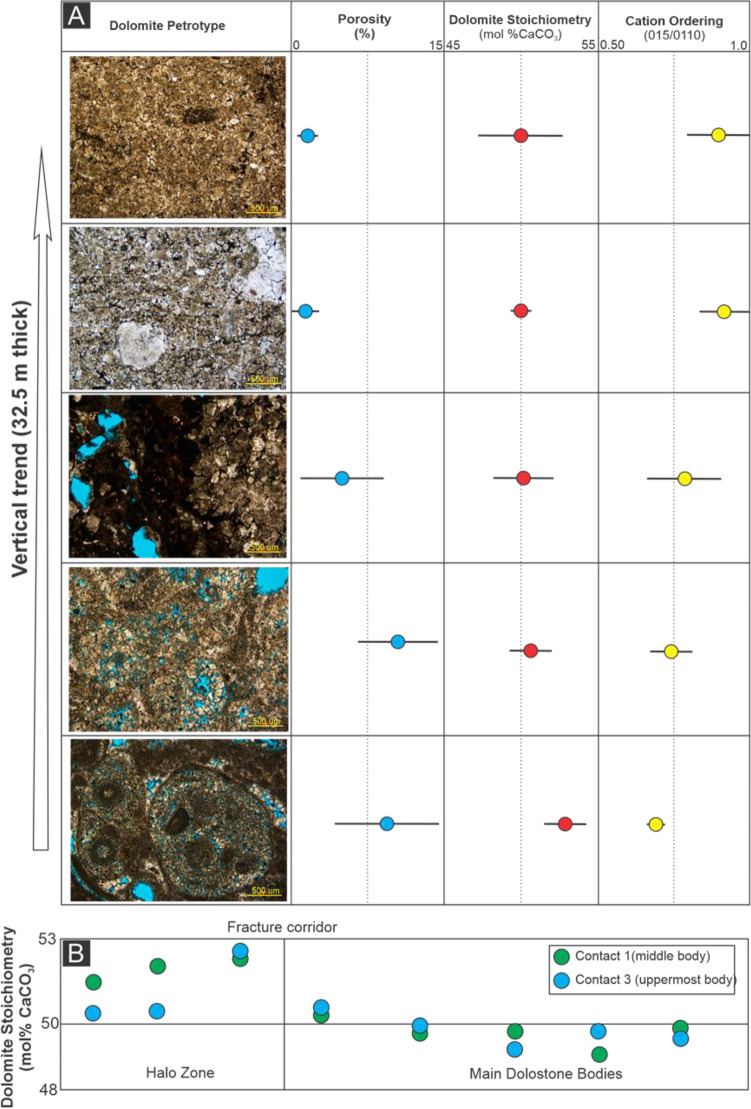
(**A**) Thin section images showing apparent changes in dolomite fabrics from FP (lower interval) to FD (upper interval). Overall, porosity decreases and dolomite stoichiometry-ordering increase upward. (**B**) A consistent pattern of less stoichiometric dolomite towards the dolomitization fronts and halos.

Overall, the two lowermost FP dolomite bodies show horizontal and vertical diffuse diagenetic contacts. Thick zones (up to 10 m wide) of partially replaced limestone occur at the transition from dolomite to limestone and are hereafter referred to as halo zones. The absence of a distinct colour contrast between the dolomite and limestone makes it difficult to precisely map the reaction front size and geometry in outcrop. The FP dolomite comprises euhedral to subhedral crystal textures with unimodal crystal sizes (30–100 µm) and a higher porosity (m = 8.9 ± 3.2%) than the adjacent limestone (m = 1.6 ± 0.9%; Fig. 3A). The middle to upper intervals of the section exhibit a distinct colour contrast between grey limestone and dark brown dolomite, with three separate bed-perpendicular terminations bounded by fracture corridors (Fig. 2B). The latter exhibit thin halo zones (5–100 cm wide) (Fig. 2B). The FD dolomite has interlocking, subhedral to anhedral fabrics with a bimodal crystal size distribution (60–420 µm) (Fig. 3A). Overall, porosity of the FD dolomite is lower than FP dolomite and decreases upwards in the succession, from m = 7.8 ± 2.9% to m = 2.9 ± 3.4% (Fig. 3A). Dolomite cement patchily occurs within the fracture corridors and less porous dolomite occurs in proximity to the fracture zone, m = 1.9 ± 0.5% near the fracture corridor and m = 7.4 ± 1.5% 40 m away from it.

**Figure 4:**
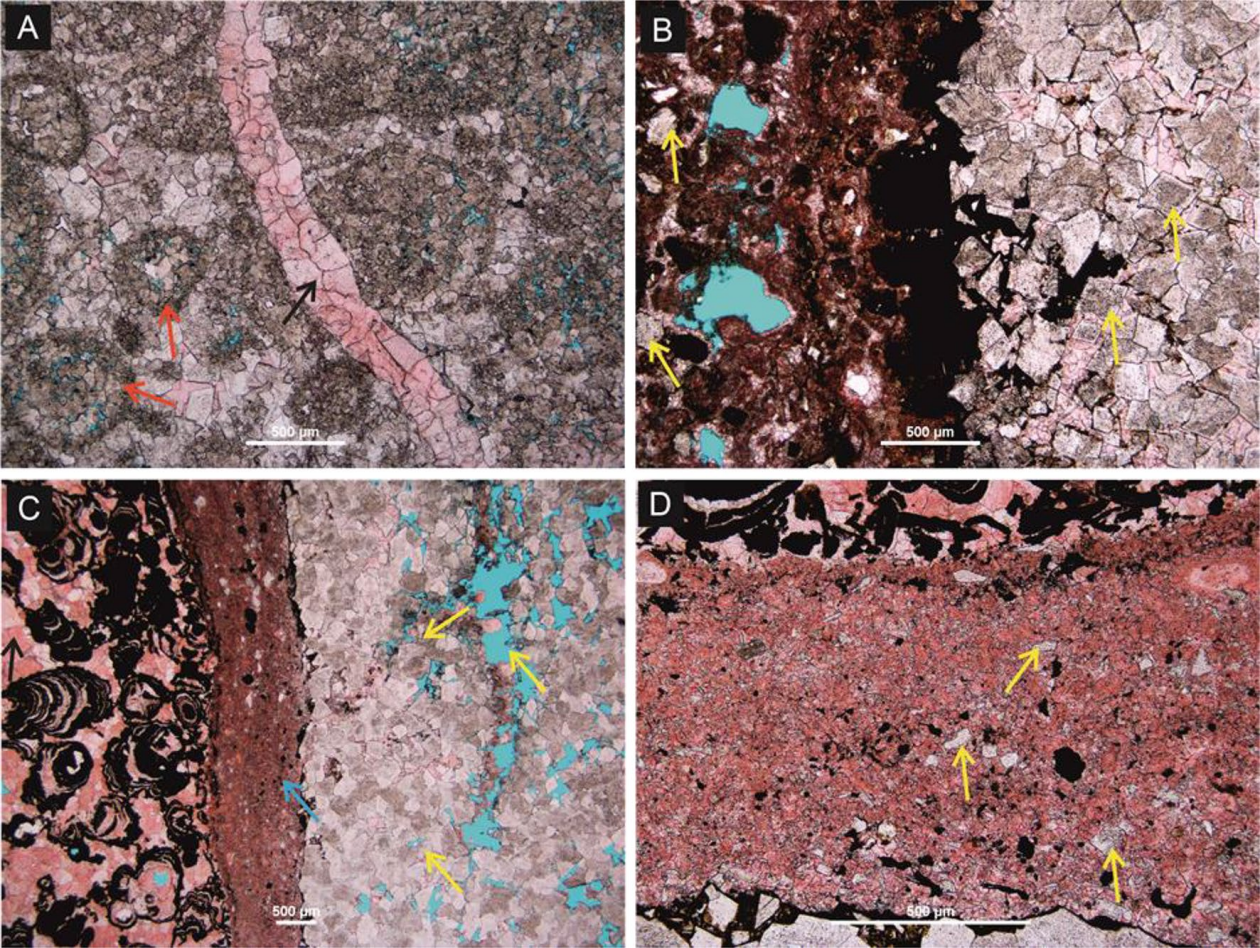
Petrographic images of different microscopic textures and fabrics observed in the Lower Jurassic carbonate, EAB. (**A**) Fracture cross cuts ooid packstone-grainstone facies, filled by blocky calcite cement (black arrow). Note the ooids were pervasively replaced by dolomite (red arrow) (**B**) Termination of dolomite fronts to mud-dominated limestone. Note the presence of isolated dolomite rhombs in the host limestone (yellow arrow). (**C**) Fracture-bounded dolomite fronts (yellow arrows) with sharp termination. The fracture is filled by fine-grained sediments (blue arrow). Blocky calcite cement indicated by a black arrow. (**D**) A close-up view of the sediment infill reveals the occurrence of dolomite rhombs and fragments within the fracture zone (yellow arrows).

In outcrop, two main fracture sets were observed: 335°–355°, n = 45 and 90° to 105°, n = 11, perpendicular and parallel, respectively, to the axis of the anticline (ENE-WSW) (Fig. 2A). Fracture density is higher in the dolomite (8–14 fractures/m) compared to the limestone (5–9 fractures/m) (Fig. 2B). Three spatially distinct NNW-SSE trending fracture corridors occur, varying from 2 to 10 m in width and approximately 25 m in length (Fig. 2B). They contain poorly sorted (0.4–6 cm), angular to sub-rounded fragments of dolomite and limestone with minor, inter-clast sandstone and calcite cement. The fracture filling calcite is characterized by blocky crystals with unimodal size distribution (200–300 µm) (Fig. 4A).

Downward tapering fissures, up to 5 m deep, with a maximum width of 10 m, penetrate downwards from the uppermost layer, localising at the intersection of the two main fracture sets. These fissures contain poorly sorted clasts (ranging from < 1 cm to > 10 cm in diameter). The largest clasts are angular, and form a mosaic breccia, whilst the smaller clasts are sub-spherical, rounded and chaotic (Fig. 2B). The clasts are all supported by a matrix of red, very fine-grained sandstone to siltstone, and cemented by calcite. The fissures pass downwards into irregularly shaped vertical pipes, < 1 m wide, filled by a sandstone-supported carbonate breccia. At the microscopic scale, the dolomite-limestone transitions appear have two different types of termination: (1) diffuse, when terminated into a fine-grained mudstone to wackestone limestone (Fig. 4B) and (2) sharp, when bounded by fractures filled by sediments (Fig. 4C, D). This is common when the adjacent host rock is grain-dominated or crystalline limestone. (Fig. 4C, D).

### Mineralogy and isotopic composition

The dolomite in the lowermost and lower to middle intervals is non-stoichiometric and poorly ordered, with average stoichiometry and cation ordering of 52.9 ± 1.1 mol% CaCO_3_ and 0.72 ± 0.03, respectively (Fig. 3A). The average stoichiometry of dolomite in the middle to upper intervals is 50.5 ± 1.8 mol% CaCO_3_ and the average cation ordering is 0.82 ± 0.09 (Fig. 3A; see supplementary material). As well as an upward-increase in stoichiometry and ordering, a lateral trend of decreasing dolomite stoichiometry (more calcium-rich) is observed beyond the fracture corridor and into the partially dolomitized halos of the middle to uppermost bodies (Fig. 3B). The cation ordering, however, does not show any systematic lateral trend.

**Figure 5:**
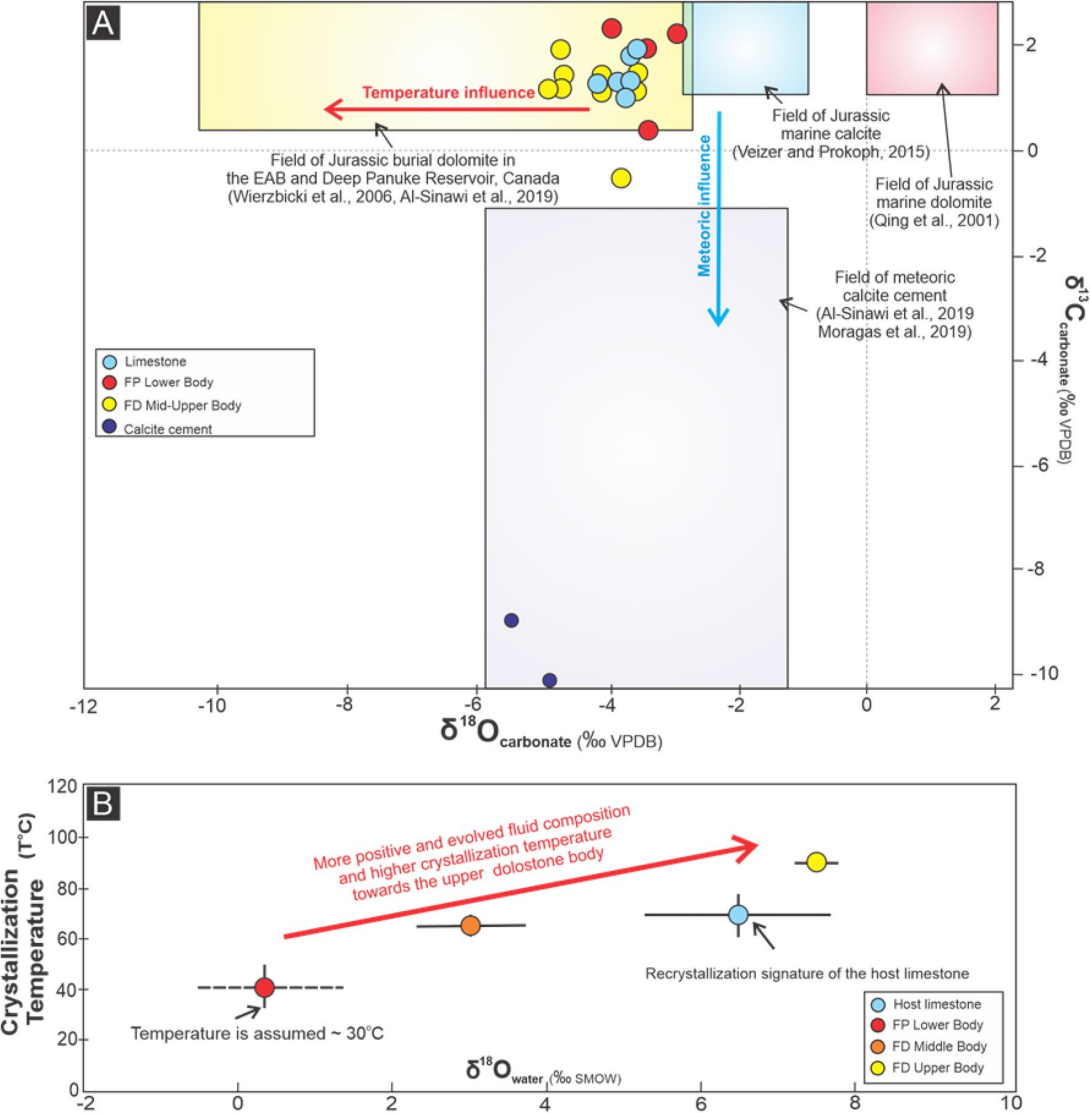
(**A**, **B**) Cross plots between δ^18^O and δ^13^C, and δ^18^O_water_ and ∆_47_-derived temperature. Overall, the isotopic values of FP and FD dolomites are comparable and lighter than limestone, respectively^39,42^. A clear trend of more evolved fluid and hotter temperature towards the upper interval.

In the limestone, the range and average values of both δ^13^C (m = 1.4 ± 0.3‰) and δ^18^O (m = − 3.7 ± 0.4‰) values are slightly more enriched in δ^18^O than the dolomite (Fig. 5A; see supplementary material). Two different calcite cements, one white and one black, exhibit a comparable range of δ^18^O values, from − 5.2 to − 4.2‰ (m = − 4.7‰) and δ^13^C values, from − 10.1 to − 8.6‰ (m = − 9.3‰). Although there is no obvious differentiation between δ^13^C values in FP and FD dolomite, δ^18^O_dolomite_ values are slightly higher (m = − 3.5 ± 0.5‰) in FP compared to FD dolomite (m = − 4.3 ± 0.5‰) (Fig. 5A; see supplementary material). The average clumped isotope (∆_47_) derived, temperatures and δ^18^O_water_ values can be divided into three groups: (1) 0.501‰, 66 °C ± 3.9 °C and 3.1 ± 0.71‰ SMOW for the middle FD dolomite; (2) 0.456‰, 90.0 °C ± 1.4 °C and 7.5 ± 0.28‰ SMOW for the FD dolomite in the uppermost interval; and (3) 0.493‰, 69 °C ± 8.5 °C and 6.5 ± 1.2‰ SMOW for the limestone (Fig. 5B; see supplementary material).

## Discussion

### Fracture system

In the studied succession, open-mode fractures are principally oriented perpendicular to subparallel (NW–SE) and parallel (E-W) to the anticlinal axis. Extensional fractures commonly form parallel to the axis of anticlines, around anticlinal hinges, during flexure^32–34^, suggesting a genetic relationship between the formation of the Amsittene Anticline and fracturing. The Amsittene Anticline is interpreted to have grown from the Middle Jurassic, initiated by salt diapirism^14,35^. It continued to form during basin inversion and exhumation in the Late Jurassic to Cretaceous with further growth and tightening during the Cenozoic (Eocene to Recent) Alpine Orogeny^36^. It is not possible from the current study to assess with confidence whether salt diapirism or compression controlled the formation of the fracture corridors, but their orientation brackets the timing of their formation to post middle Jurassic.

### Genesis of dolomite

The stratabound geometry and euhedral to subhedral, zoned dolomite crystals that characterize FP dolomite are indicative of a low temperature dolomite formed from seawater^1,2,37,38^. The poor stoichiometry and ordering of the dolomite are also suggestive of early-formed, low temperature dolomitization^39^. No clumped isotope data were available for these beds, but assuming a slightly elevated sea surface temperature (at least 30 °C) and elevated seawater temperature at shallow depths in this basin associated with post-rift thermal subsidence^40,41^, as it has been reported in the adjacent high Atlas basin^40,41^, then the calculated δ^18^O_water_ is within the range of Jurassic seawater^42^ (Fig. 5B and see supplementary material). Under these conditions, precipitation of dolomite at temperatures of up to 40 °C could have been achieved by reflux of seawater to depths of up to 500 m beneath the surface or near-surface thermal anomaly due to salt diapirism. In both scenarios, the calculated δ^18^O_water_ still fall within the expected range of Jurassic seawater (Fig. 5B and see supplementary material). It is also possible that there was a thermal anomaly within the Arich Ouzla Formation caused by the high thermal conductivity of the underlying salt, which would have increased formational temperatures and facilitated dolomitization.

The FD dolomite in the middle to upper beds is characterized by (1) subhedral to anhedral, stoichiometric and well-ordered crystals; (2) depleted δ^18^O_dolomite_ values; (3) moderately high crystallization temperatures (65.5–90.1 °C) and heavy δ^18^O_water_ (3.5–7.1‰ SMOW) (Fig. 5B) compared to the FP dolomite. The typically unzoned, stoichiometric and ordered dolomite, with non-planar crystal textures, implies recrystallization and stabilization of earlier formed FP dolomite^39,43^. The upward-increase in stoichiometry and cation ordering, and associated decrease in porosity implies fluids flowed up the corridors and outwards, perhaps beneath a now-eroded low permeability layer of Amsittene Formation. With a maximum burial depth of 1.5 km^11^ and a maximum geothermal gradient of 30 °C/km^17^, then the maximum burial temperature of the succession would be 65 °C (assuming a seawater temperature of 20 °C). Since the highest measured temperature (90 °C) would require burial of the formation to up to 3 km depth, it is highly likely that fluids were hydrothermal. The depleted δ^18^O values of the FD dolomites when compared to other dolomite fabrics can therefore be explained by recrystallization by warm dolomitizing fluids^44^, consistent with other dolomite bodies formed in Jurassic carbonates elsewhere in the Agadir–Essaouira Basin and the neighbouring basins that are interpreted to be hydrothermal^41,44–46^ (Fig. 5A). The fluid source cannot be fully constrained, but could be explained by deep convection of seawater along faults and fractures with some degree of modification through interactions with the underlying Triassic sandstone, salt and the CAMP basalt^44^. The presence of thick salt unit could also have facilitated fluid convection because of the temperature gradient created by its high thermal conductivity; such a process has been invoked in several salt-dominated basins^47–50^.

### Termination of dolomite

**Figure 6:**
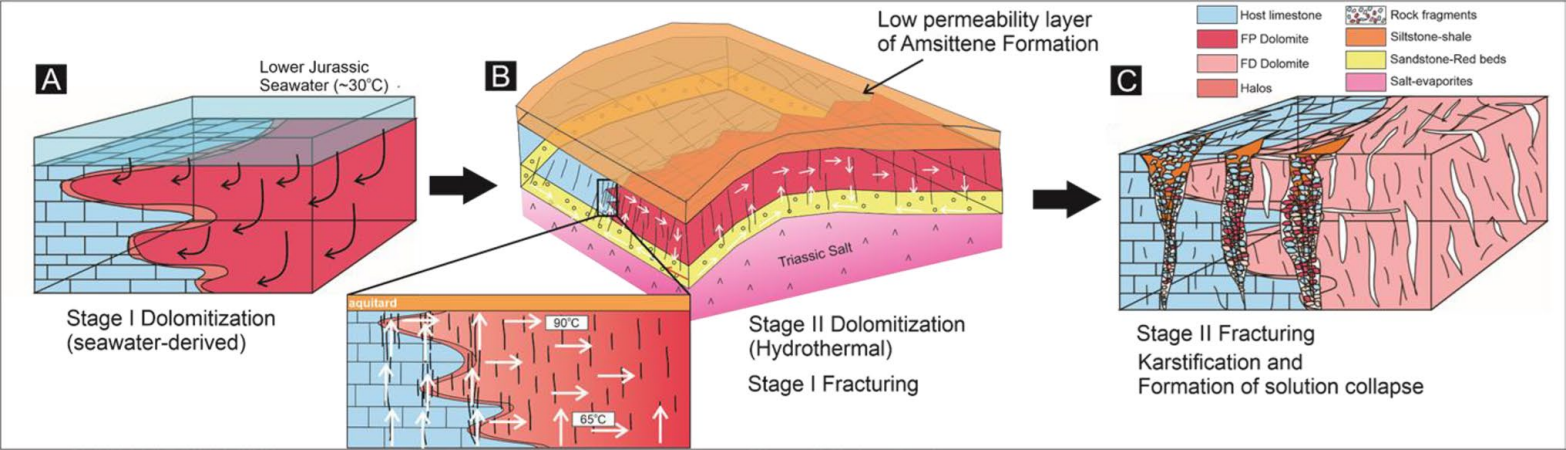
Conceptual model on the evolution of dolomitization, fracture system and karstification in the study area. (**A**) Early, seawater-derived dolomitization which predated fracturing. (**B**) Formation of fracture corridors, preferentially along the dolomite-limestone boundary. This provides vertical fluid pathways for upward flux of warm, Mg-rich fluids. (**C**) The last stage represents the development of solution collapse during the interaction between groundwater/meteoric water and carbonate rocks associated with the formation of Cenozoic Alpine Orogeny.

The transition between FP dolomite and limestone, in the lower to middle stratigraphic interval, is gradual and diffuse and within beds. Given this, and the poor stoichiometry and ordering of the dolomite at the reaction fronts, the first phase of dolomitization is interpreted to have terminated as a result of a decrease in the dolomitization potential or capacity of the dolomitizing fluid^5,6^ rather than as a result of a change in rock properties or stratal architecture (Fig. 6A). This is evident from the Mg/Ca ratio profile which decreases across the dolostone bodies into the limestone (Figs. 3A, B, 6A). Previous studies have also indicated gradual, diffuse termination is commonly associated with changes in the fluid properties^2,5^.

The location of fracture corridors at the apparent contact between FP dolomite and limestone could be interpreted to record a failure of dolomitizing fluids to migrate across the fracture corridors (i.e. the corridors acted as lateral barriers to fluid flow). However, the fractures within the corridors are open and FP dolomite is found in small volumes beyond the fracture corridor, whereas FD dolomite is not. In such a case, the hydrothermal dolomitizing fluids were vented upwards along these fracture corridors^4,5,46,51–54^. This is further corroborated by the presence of (1) fragments of FP dolomite within fracture corridors and (2) non-stoichiometric and poorly ordered dolomite in the adjacent limestone and halo zones, beyond the fracture corridors (Fig. 4A, D). These suggest that FP dolomitization predated fracturing and the fracture corridors formed after dolomitization because of the mechanical contrast between dolomite (high bulk and Young’s modulus) and limestone (low to intermediate bulk and Young’ modulus) (Fig. 6B). It is not possible from the current study to assess with confidence whether salt diapirism or compression controlled the formation of the fracture corridors, but previous work shows the salt diapirism occurred in the late Triassic and continued during the Jurassic^54^. These salt diapirs created localized topography that allowed enhanced fluid flow and created the FP dolomite. Further progression of this salt diapir could have a pronounced control on the formation of FD dolomite through fracture-controlled hydrothermal dolomitization.

Either way, fracturing led to the mosaic brecciation of the precursor dolomite and created a network of open fractures which could have later acted as vertical fluid pathways for warm, magnesium-rich fluids, forming FD dolomite. The uppermost FD dolomite appears to be crosscut by two fracture corridors (Fig. 2B), suggesting either that fracturing post-dated formation of the FD dolomite or that fluids flowed upwards and away from the fracture corridor to recrystallize the FP dolomite to form stoichiometric and ordered FD dolomite (Fig. 2). Rapid and continuous fluid flux led to recrystallization of the precursor FP dolomite in proximity to the fracture corridor in the middle and upper beds, perhaps because fluids were channelled laterally when the fractures terminated beneath the now-eroded, low permeability strata. Dolomitization took place quickly, so that thermal re-equilibration did not occur and the FD records evidence of hydrothermal fluid flow. The presence of dolomite cement and less porous dolomite in proximity to the fracture zone, with an increase in porosity away from the fracture corridors, suggests that overdolomitization subsequently took place in the vicinity of the fractures, limiting the outward flux of dolomitizing fluids, forming a retreating dolomitization front^5^ (Fig. 6A, B).

### Karstification

In outcrop, the fracture corridors are overprinted by fissures filled by fragments of carbonate and siliciclastic rocks. These downward-tapering fissures have irregular margins, typical of dissolution, particularly at their base, suggesting that they formed by dissolution. Their downward-tapering morphology is consistent with downward fluid-flux, such as would occur during karstification by groundwater percolation and soil processes. Their occurrence along fracture corridors indicates that the fractures provided a permeable pathway for the ingress of the fluids; the locus of the fissures at the intersection of fracture corridors suggests that these zones were exploited as they had the highest vertical permeability (Fig. 6C). The largest, fitted clasts are probably remnants of the mosaic breccia with the fracture corridor, whilst the smaller clasts are interpreted to have formed by the dissolution and collapse of this mosaic breccia (Fig. 6C). The fissures were then filled by sandstone and siltstone. Although it is not possible to more precisely date the sediments, the red colour of the siltstone implies subaerial conditions, whilst the preservation of lamination is indicative of aqueous flow. This information brackets the timing of these fissures to, at the latest, the Oligo-Miocene, and the sediments themselves resemble Late Eocene-aged siliciclastic sediments of Im-n-Tanout Formation^11^. The potential for near surface fissures to remain open^55^ means that they could have been long-lived features. This argument is further supported by the presence of calcite cements with typically low δ^18^O values (up to − 5‰ VPDB) and δ^13^C values (up to − 10‰ VPDB), characteristic of meteoric diagenesis (Fig. 5B) whilst REE analysis^44^ has measured a Y/Ho ratio of 27.5–39.9 (m = 33.7)^56^, which is typical of meteoric fluids (seawater Y/Ho = 48–78)^56^.

## Implications and conclusions

Dolomitization fronts inform our understanding of how metasomatic reactions proceed in time and space since the termination of replacement and formation of the reaction front indicates a change in physio-chemical conditions. Although transitions might appear sharp in outcrop, detailed petrographic and geochemical analysis reveals that they are not simple, but the result of multiple, potentially related events. Poorly ordered, non-stoichiometric dolomite that forms in near surface settings is recrystallized^39^, either downwards in refluxing systems^57^ or laterally, from faults. This can result in a back-stepping of the reaction front in which the reaction fronts retreating closer to the fluid source or fracture corridors as porosity is progressively occluded through subsequent dolomitization^5^.

This study shows that the mechanical contrast created at the reaction front led to preferential fracturing during anticlinal growth. This fracture pathway subsequently facilitated an upward-flux of fluids that overprinted the early-formed dolomite, promoted by an increased heat flow above the underlying Triassic salt. As dolomitization proceeded, dolomite cement was precipitated within the fracture corridors and fluid flux was terminated—another example of the back-stepping of the reaction front. With further flexure, uplift and erosion, the fracture corridors then acted as a conduit for groundwater or meteoric water, creating karstic fissures that became infilled by collapsed carbonate and sediment. Overall, therefore, it can be shown that the inheritance of prior metasomatic processes governed rock deformation and then, subsequently fluid flow and enhanced rock-fluid interaction. In the near surface, such patterns of inheritance have implications to the prediction of geohazards, such as sink-holes. In the subsurface, such contacts might create significant perturbations to the flow of water, gas or hydrocarbons. It has been shown that even an apparently simple natural reaction front records a complex, multiphase, history of recrystallization and deformation.

## Supplementary Information

Supplementary Information.

## Data Availability

The datasets used and/or analysed during the current study available from the corresponding author on reasonable request. Some datasets analysed during this study are included in this published article and its supplementary information file.
