# Supplementary material for: Coevolution of diagenetic fronts and fluid-fracture pathways
Source: Sci Rep. 2022 Jun 3;12:9278. doi: 10.1038/s41598-022-13186-1 (PMC9166768; doi:10.1038/s41598-022-13186-1)
Supplement: Supplementary file 1 — Supplementary Information. [file 41598_2022_13186_MOESM1_ESM.docx]

**Supplementary Material**

**Table 1. Results from limestone and dolomite mineralogical properties and isotopic analyses.**

| **Phase** | **Dolomite Stoichiometry (mol % CaCO3** | | **Dolomite Cation Ordering (015/110)** | | **Thin Section Porosity** | | **^13^C_VPDB_ (‰)** | | **^18^O_VPDB_ (‰)** | | **Δ47-RF (CDES @ 90^o^C)** | | **TΔ_47_ (^o^C)^a^** | | **^18^O_water_ (‰, SMOW)** | |
| --- | --- | --- | --- | --- | --- | --- | --- | --- | --- | --- | --- | --- | --- | --- | --- | --- |
| **Host Limestone** | |  | |  | |  | |  | |  | |  | |  | |  |
| Min |  |  | | 0.6 | | 1.1 | | -4.1 | | 0.493 ± 0.016 | | 69 ± 8.5 | | 6.5 ± 1.2 | |  |
| Mean |  |  | | 1.6 | | 1.4 | | -3.7 | |  |  |  |  |  |  |  |
| Max |  |  | | 3.5 | | 1.8 | | -4.6 | |  |  |  |  |  |  |  |
| SD |  |  | | 0.9 | | 0.3 | | 0.4 | |  |  |  |  |  |  |  |
|  |  |  | |  | |  | |  | |  | |  | |  | |  |
| **Fabric Preserving Dolomite (Lowermost and Lower to Middle)** | | | | | |  | |  | |  | |  | |  | |  |
| Min | 51.7 | 0.67 | | 2.8 | | 1.9 | | -4.1 | |  | |  | | 0.32 ± 0.78 (temperature is assumed @35-50 ^o^C) | |  |
| Mean | 52.9 | 0.72 | | 8.9 | | 2 | | -3.5 | |  | |  | |  |  |  |
| Max | 54.4 | 0.80 | | 13.7 | | 2.1 | | -2.8 | |  | |  | |  |  |  |
| SD | 1.1 | 0.03 | | 3.2 | | 0.1 | | 0.5 | |  | |  | |  |  |  |
|  |  |  | |  | |  | |  | |  | |  | |  | |  |
| **Fabric Destructive Dolomite (Middle and Middle to Upper)** | | | | | |  | |  | |  | |  | |  | |  |
| Min | 48.3 | 0.63 | | 2.5 | | -0.7 | | -4.8 | | 0.501 ± 0.008 | | 66 ± 3.9 | | 3.1 ± 0.71 | |  |
| Mean | 50.5 | 0.82 | | 7.8 | | 0.9 | | -4.3 | |  |  |  |  |  |  |  |
| Max | 52.7 | 1.00 | | 14.2 | | 1.8 | | -3.5 | |  |  |  |  |  |  |  |
| SD | 1.8 | 0.09 | | 2.9 | | 1.0 | | 0.5 | |  |  |  |  |  |  |  |
|  |  |  | |  | |  | |  | |  | |  | |  | |  |
| **Fabric Destructive Dolomite (Uppermost)** | | | |  | |  | |  | |  | |  | |  | |  |
| Min | 47.8 | 0.80 | | 0.6 | | 1.1 | | -3.3 | | 0.456 ± 0.002 | | 90 ± 1.4 | | 7.5 ± 0.28 | |  |
| Mean | 50.2 | 0.90 | | 2.9 | | 1.1 | | -3.1 | |  |  |  |  |  |  |  |
| Max | 52.4 | 1.00 | | 9.3 | | 1.2 | | -3.0 | |  |  |  |  |  |  |  |
| SD | 1.7 | 0.08 | | 3.4 | | 0.0 | | 0.1 | |  |  |  |  |  |  |  |

^a^ Following the temperature conversion equation of Staudigel et al. (2018)

| Types | Replacive Dolomite (Amsittene) | | | |  | Host Limestone (Amsittene) | | |  |  | Replacive Dolomite (Amsittene) | | | |
| --- | --- | --- | --- | --- | --- | --- | --- | --- | --- | --- | --- | --- | --- | --- |
| Run # | 1 | 2 | av | sd |  | 1 | 2 | av | sd |  | 1 | 2 | av | sd |
| Run ID | C07545 | C07568 |  |  |  | C07544 | C07646 |  |  |  | C07665 | C07671 |  |  |
| Sample | AML-D1C | AML-D1C |  |  |  | AML-L1C | AML-L1C |  |  |  | AML-D3C | AML-D3C |  |  |
| Date | 12/25/18 | 12/29/18 |  |  |  | 12/25/18 | 12/27/2019 |  |  |  | 01/14/19 | 01/15/19 |  |  |
| Block | 6 | 6 |  |  |  | 5 | 5 |  |  |  | 5 | 3 |  |  |
| CC | 14.5 | 14.5 |  |  |  | 14.5 | 14.5 |  |  |  | 14.5 | 14.5 |  |  |
|  | Iterative | Iterative |  |  |  | Iterative | Iterative |  |  |  | Iterative | Iterative |  |  |
| δ13C | 1.280746 | 1.232222 | 1.26 | 0.02 |  | 0.977736 | 0.984408 | 0.98 | 0.003 |  | 1.112459 | 1.135806 | 1.124133 | 0.009531 |
| Carbonate | -3.09494 | -3.253 | -3.2 | 0.06 |  | -3.8307 | -3.631 | -3.7 | 0.083 |  | -2.21878 | -2.43689 | -3.01878 | 0.337666 |
| δ47CO2 | 23.2553 | 23.061 |  |  |  | 22.17307 | 22.42505 |  |  |  | 23.98287 | 23.78464 |  |  |
| Δ47CO2 | -0.34979 | -0.33275 |  |  |  | -0.36999 | -0.33238 |  |  |  | -0.38312 | -0.37849 |  |  |
|  | 0 | 0 |  |  |  | 0 | 0 |  |  |  | 0 | 0 |  |  |
| Heated Slope | 0.004275 | 0.004275 |  |  |  | 0.004275 | 0.004275 |  |  |  | 0.004275 | 0.004275 |  |  |
| 25C Slope | 0.003032 | 0.003194 |  |  |  | 0.003032 | 0.003559 |  |  |  | 0.003559 | 0.003559 |  |  |
| 50C Slope | 0.002344 | 0.002351 |  |  |  | 0.002344 | 0.002361 |  |  |  | 0.002361 | 0.002361 |  |  |
| Slope Average | 0.003217 | 0.003273 |  |  |  | 0.003217 | 0.003398 |  |  |  | 0.003398 | 0.003398 |  |  |
| Heated Intercept | -0.85698 | -0.85698 |  |  |  | -0.85698 | -0.85698 |  |  |  | -0.85698 | -0.85698 |  |  |
| 25 C Intercept | -0.01921 | -0.02207 |  |  |  | -0.01921 | -0.02851 |  |  |  | -0.02851 | -0.02851 |  |  |
| 50C Intercept | -0.13359 | -0.1373 |  |  |  | -0.13359 | -0.14193 |  |  |  | -0.14193 | -0.14193 |  |  |
| Heated Reference | 0.026624 | 0.026624 |  |  |  | 0.026624 | 0.026624 |  |  |  | 0.026624 | 0.026624 |  |  |
| 25C Reference | 0.925203 | 0.925203 |  |  |  | 0.925203 | 0.925203 |  |  |  | 0.925203 | 0.925203 |  |  |
| 50C Reference | 0.80495 | 0.80495 |  |  |  | 0.80495 | 0.80495 |  |  |  | 0.80495 | 0.80495 |  |  |
| ETF Slope | 1.073778 | 1.078109 |  |  |  | 1.073778 | 1.085996 |  |  |  | 1.085996 | 1.085996 |  |  |
| ETF Intercept | 0.94702 | 0.950839 |  |  |  | 0.94702 | 0.957519 |  |  |  | 0.957519 | 0.957519 |  |  |
| Δ47-[SGvsWG]0 | -0.4246 | -0.40824 |  |  |  | -0.44132 | -0.40858 |  |  |  | -0.46462 | -0.45932 |  |  |
| Δ47-RF | 0.491094 | 0.510713 |  |  |  | 0.473145 | 0.513801 |  |  |  | 0.452943 | 0.4587 |  |  |
|  | 0 | 0 |  |  |  | 0 | 0 |  |  |  | 0 | 0 |  |  |
| Δ47-AC (+0.092) (Henkes et al. (2013) | 0.583094 | 0.602713 |  |  |  | 0.565145 | 0.605801 |  |  |  | 0.544943 | 0.5507 |  |  |
| Temp. (Eq. 9) Calibrated 0-50C | 55.78951 | 50.43373 |  |  |  | 60.92963 | 49.61416 |  |  |  | 67.01382 | 65.24639 |  |  |
| Temp. (Eq. 10) Calibrated 7-77C | 79.49503 | 68.18034 |  |  |  | 90.89684 | 66.49638 |  |  |  | 105.1556 | 100.9229 |  |  |
| SIL Temperature | 70.33911 | 60.64961 | 65.49 | 3.96 |  | 79.98497 | 59.19762 | 69.59 | 8.49 |  | 91.87875 | 88.36786 | 90.12331 | 1.433315 |
| Temp. (Eq. 9) Kelvin | 343.4552 | 333.931 |  |  |  | 352.9223 | 332.3476 |  |  |  | 365.0287 | 361.5179 |  |  |
| Calcite Oxygen (SMOW) | 27.71939 | 27.55645 |  |  |  | 26.96089 | 27.16677 |  |  |  | 28.62264 | 28.39778 |  |  |
| Alpha (Kim and O'Neill 1997) | 1.020279 | 1.021808 |  |  |  | 1.018843 | 1.02207 |  |  |  | 1.017118 | 1.017606 |  |  |
| Alpha (Oneil 1969) | 1.02089 | 1.022283 |  |  |  | 1.019618 | 1.022526 |  |  |  | 1.018134 | 1.018549 |  |  |
| Water Kim & O'Neill (1997) | 7.292694 | 5.626179 |  |  |  | 7.967688 | 4.986264 | 6.48 | 1.21 |  | 11.31078 | 10.60482 |  |  |
| WATER Oneil et al (1969) | 6.689565 | 5.158628 |  |  |  | 7.202063 | 4.538242 |  |  |  | 10.30156 | 9.669525 |  |  |
| Alpha (Horita 2014) | 1.023751 | 1.025357 |  |  |  |  |  |  |  |  | 1.020635 | 1.021105 |  |  |
| Alpha (Matthew and Katz 1973) | 1.022955 | 1.024519 |  |  |  |  |  |  |  |  | 1.019921 | 1.020378 |  |  |
| Dolomite Fluid (Horita 2014) | 3.876055 | 2.144797 | 3.01 | 0.706 |  |  |  |  |  |  | 7.825688 | 7.14205 | 7.483869 | 0.279094 |
| Dolomite Fluid (Matthew and Katz 1973) | 4.657428 | 2.964877 |  |  |  |  |  |  |  |  | 8.53181 | 7.859503 |  |  |
